# A small-scale bacterial-based liquid Culture Method for *Steinernema hermaphroditum*

**DOI:** 10.17912/micropub.biology.001549

**Published:** 2025-04-28

**Authors:** Nathan Y. Rodak, Chieh-Hsiang Tan, Paul W. Sternberg

**Affiliations:** 1 Division of Biology and Biological Engineering, California Institute of Technology, 1200 East California Boulevard, Pasadena, CA, USA; 2 Current Address: Case Western Reserve University, Cleveland, OH, USA

## Abstract

Entomopathogenic nematodes (EPN) infect and kill their insect host with the help of symbiotic bacteria. The only known hermaphroditic (androdiecious) EPN, the clade IV *Steinernema hermaphroditum* , offers opportunities for exploring both parasitic and mutualistic symbiosis, as well as for evolutionary and developmental studies. Experimental and genetic analysis of this animal is now facilitated through the development of forward and reverse genetic tools and improved culturing techniques. Here, we describe a liquid-culture technique adapted for this worm. The method can be a starting point for the development of large-scale cultivation of the worm and provides a method to generate infective juveniles without an insect host and either with or without its native symbiotic bacteria.

**Figure 1. A small-scale bacterial-based liquid Culture Method for Steinernema hermaphroditum:**
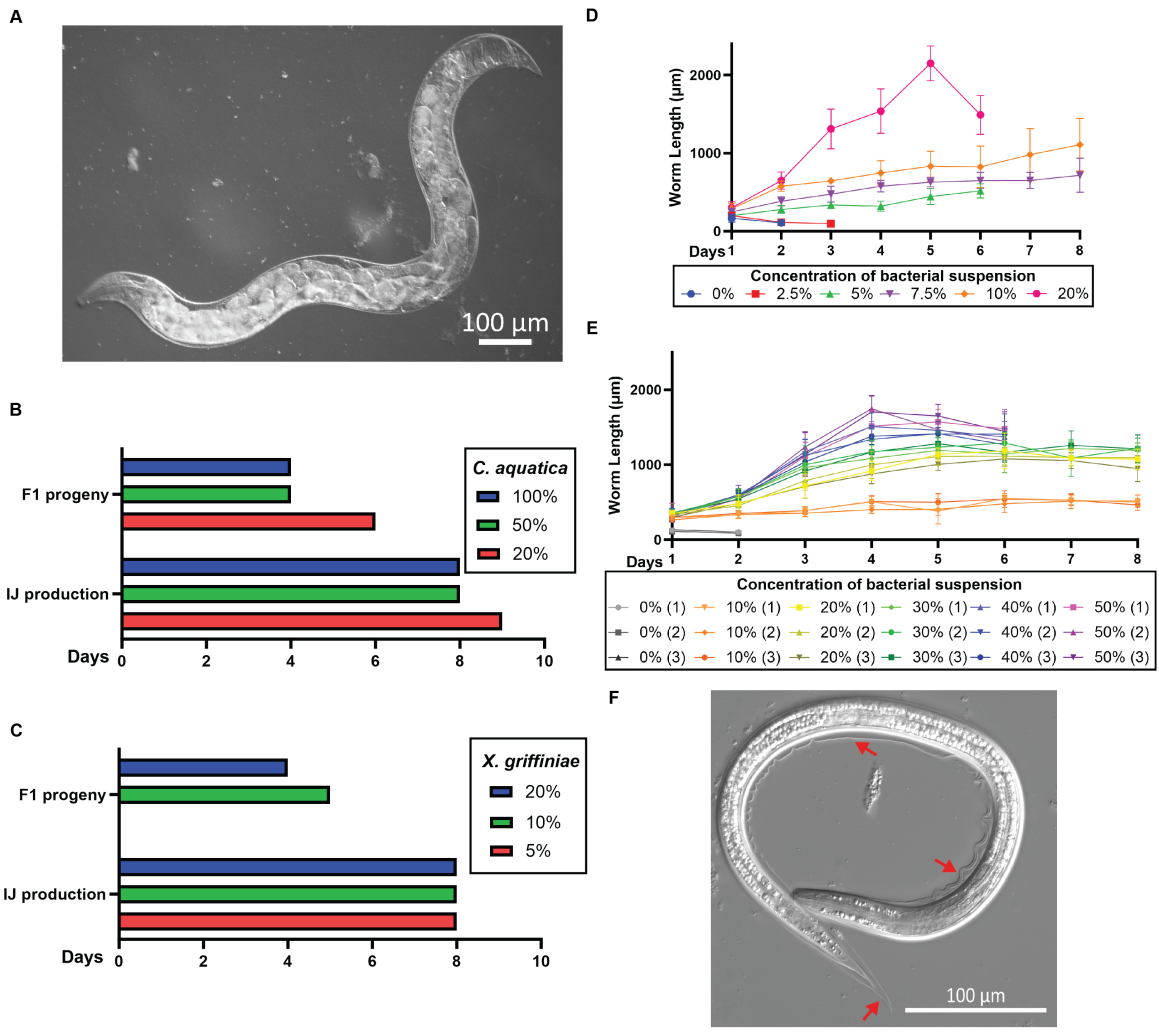
(A) A gravid adult *S. hermaphroditum* grown at 25°C in S Medium liquid culture using *C.* aquatica as a food source. Left: anterior; down: ventral. Scale bar, 100 µm. (B, C) Bar charts showing the number of days required for P _0_ embryos to develop into adults and produce F _1_ larvae (upper charts, the day when the first F _1_ larvae appear) and for the development of infective juveniles (IJs) (lower charts, the day when the first IJs appear). Three independent populations of worms were assayed for each condition which gives the same results. (B) Liquid culture in S Medium containing 20%, 50%, or 100% of concentrated *C. aquatica* suspension in S Medium by volume. (C) Liquid culture in S Medium containing 5%, 10%, or 20% (by volume) of concentrated *X. griffiniae* suspension in S Medium. Worms failed to produce progeny when grown in 5% *X. griffiniae* . (D, E) Worms grow faster in cultures with a higher concentration of food. Average lengths of worms grown in liquid culture containing varying concentrations of *C. aquatica* suspension. (D) 0% - 20% of concentrated *C. aquatica* by volume. Values are mean ± SD (n= 8 - 50). (E). 0% - 50% of concentrated *C. aquatica* by volume. Data from three independent populations are presented separately. Values are mean ± SD (n = 5 - 15, 15 worms were measured in most data points). (F) An infective juvenile (IJ) stage *S. hermaphroditum* from a S Medium / *C. aquatica* liquid culture incubated at 25°C. The worm has a sealed mouth, a constricted pharynx, and the entire body is enclosed by a seemly detached second larval stage (J2) cuticle (red arrows) that is highly visible. Scale bar, 100 µm.

## Description

A liquid-based culturing system for small animals such as *Caenorhabditis elegans* (Maupas 1900) is helpful in that it provides not only a method for the mass production of the animal but also is a valuable research tool, providing a uniform environment as a population grows. Liquid culture has been useful in the study of aspects of *C. elegans* biology, including stress resistance, induction of dauer larvae, and lifespan (Boyd et al. 2003; Baugh et al. 2011; Park et al. 2017; Hibshman et al. 2021). *Steinernema hermaphroditum* (Stock et al. 2004) is a Clade IV nematode thatå is evolutionarily distinct from the Clade V nematodes currently used extensively in the laboratory, such as *C.* *elegans* (Corsi et al. 2015), *Oscheius tipulae* (Lam and Webster 1971; Felix 2006), and *Pristionchus pacificus* (Sommer et al. 1996; Sommer 2006). It is also the only characterized consistently hermaphroditic entomopathogenic nematode (EPN) (Cao et al. 2022). These characteristics, along with a chromosomally scaffolded annotated genome and the availability of both forward and reverse genetic techniques, establish *S. hermaphroditum* as an excellent model for studying evolution, development, mutualistic symbiosis, and parasitism (Cao et al. 2022; Cao 2023; Schwartz et al. 2024; Schwarz et al. 2025). Bacteria-based liquid cultures have been described for a number of entomopathogenic nematodes, most of which utilized their associated symbiotic bacteria as the food source (Buecher and Popiel 1989; Lunau et al. 1993; Ehlers et al. 1998; Ehlers et al. 2000; Ferreira et al. 2014; Addis et al. 2016; Ferreira et al. 2016; Pérez-Campos et al. 2018; Dunn et al. 2019). Here, we describe a liquid culture method for *S. hermaphroditum* that we adapted and modified from the method used in *C. elegans* . We showed that the worms could thrive under these conditions and that these liquid cultures can produce infective juveniles (IJs), with or without their symbiont.

We modified the S Medium-based liquid culture method widely used in *C. elegans* research (Stiernagle 2006; Hibshman et al. 2021) by replacing *Escherichia coli* with either the non-symbiont bacteria *Comamonas aquatica* (Avery and Shtonda 2003; Watson et al. 2014), or the native symbiont *Xenorhabdus griffiniae* (Tailliez et al. 2006). We showed previously that *S. hermaphroditum* could be cultured on lawns of the non-symbiotic bacteria *C. aquatica* (Cao et al. 2022; Rodak et al. 2024). When cultured on agar-based media, *C. aquatica* offers several advantages over the native symbiont *X. griffiniae* , including a higher growth rate, better transparency of the worm, and an apparent lack of phase variation observed in *Xenorhabdus* species (Akhurst 1980). We therefore adapted *C. aquatica* as the primary food source used in this study, while also developing protocols for liquid culture of *S. hermaphroditum* using its native symbiont *X. griffiniae* as the food source.

We cultured *S. hermaphroditum* on varying concentrations of *C. aquatica* or *X.* *griffiniae* starting from fertilized embryos and recorded the lengths of the worms as they grew, the amount of time they took to reach adulthood and reproduce, and the time needed for the population to produce dispersal‑stage IJ larvae. We found that at sufficiently high food concentrations (20% of concentrated *C. aquatica* suspension or 10% of concentrated *X.* *griffiniae* suspension in S medium by volume. Concentrated bacteria suspension was obtained by resuspending overnight culture in S medium 1/10 of the original volume (10X concentrated). See methods for details.) *S. hermaphroditum* can be successfully cultivated to adulthood ( Fig. 1A ) and produce progeny ( Fig. 1B and C). In multiple independent tests, we found that increasing the food concentration increased the growth rate of the worm ( Fig. 1D and E) and decreased the amount of time needed to reproduce ( Fig. 1B and C). For example, when starting with an average of 762 worms/mL (± 220, n = 6), F _1_ progeny were observed on day 6 when *C. aquatica* (concentrated) suspension was 20% of the culture medium (by volume), but on day 4 when the *C. aquatica* suspension was 50% or 100% of the culture medium ( Fig. 1B ). Similarly, in a separate experiment with *X.* *griffiniae* as food, F1 progeny were observed on day 5 when bacteria concentrations were at 10% but on day 4 at 20% ( Fig. 1C ). When food was resupplied daily (adding 25µL, 50µL, or 100µL of concentrated *C. aquatica* to the original culture), multigenerational reproduction was observed, with newly matured adults observed up until at least day 20, suggesting the feasibility of continuous culture.

Without subsequent food supplements, the worms eventually cleared the bacteria, and young larvae developed into infective juveniles (IJs) ( Fig. 1F ) at around day 8-9 ( Fig. 1B and C). We observed that the IJs of *S. hermaphroditum* are ensheathed, with the J2 cuticle clearly observable under microscopy ( Fig. 1F ). Ensheathment of IJs is characteristic of many parasitic nematodes, including EPNs (Lee 2002), but this cuticle is readily lost in the free-living nematode model *C. elegans* (Cassada and Russell 1975). Ensheathment could explain our previous observation that the sensory neurons of *S. hermaphroditum* IJs are not surface-exposed, as assessed using DiI (1,1′-Dioctadecyl-3,3,3′,3′-Tetramethylindocarbocyanine Perchlorate) staining (Garg et al. 2022).

In summary, we have developed a method for a liquid culture of *S. hermaphroditum* using either its native symbiont, the entomopathogenic bacterium *X. griffiniae* , or, as a neutral option, the soil bacterium *C. aquatica* , as a food source. We believe this small volume liquid culture method could be a useful tool for the study of *S. hermaphroditum* biology in the laboratory and as a starting point for testing methods for large volume liquid culture.

## Methods

**Solid media nematode culture**

The wild-type strain *Steinernema hermaphroditum* strain PS9179 (Cao et al. 2022), which was inbred from the isolate CS34 (Bhat et al. 2019), was cultured on Enriched Peptone Medium agar (EPM) on a lawn that had been seeded from an overnight culture of *Comamonas aquatica* as described in Rodak et al. (2024).

**Liquid culture methods**

**S Medium (base medium)**

The recipe of S Medium was based on Stiernagle (2006) and Hibshman et al. (2021) (referred to as S-complete). Briefly, for ~1 liter of the solution, add:

(1) 1 liter of S-basal solution [per 1 L: 5.9 g of NaCl, 50 mL of 1 M KH _2_ PO _4_ solution pH 6, add deionized water to 1 L and autoclave to sterilize. After autoclave, add 1 mL of Cholesterol solution (5 mg/mL in ethanol)]

(2) 10 mL of Trace metal solution [per 1 L: 1.86 g of Disodium EDTA, 0.69 g of FeSO _4_ ·7H _2_ O, 0.2 g of MnCl _2_ ·4H _2_ O, 0.29 g of ZnSO _4_ ·7H _2_ O, 0.025 g of CuSO _4_ ·5H _2_ O, add deionized water to 1 L and autoclave to sterilize. After autoclaving, store in the dark at room temperature.]

(3) 10 mL of 1M Potassium citrate pH 6 [per 1 L: 20 g of citric acid monohydrate, 293.5 g of Tri-potassium citrate monohydrate, add deionized water to 1 L and autoclave to sterilize.]

(4) 3 mL 1M CaCl _2_ solution

(5) 3 mL 1M MgSO _4_ solution

**Concentrated bacteria (Food source)**

Recently thawed *Comamonas aquatica* DA1877 (Avery and Shtonda 2003), and *Xenorhabdus griffiniae* HGB2511 (Cao et al. 2022) were streaked onto Luria-Bertani (LB) plates, grown at 37°C and room temperature respectively, and stored either at 4°C (DA1877) or at room temperature in the dark (HGB2511). To prepare concentrated bacteria as the food source, LB medium (200 mL) in Erlenmeyer flasks (1 L size) was seeded with a single picked colony of bacteria and incubated overnight at 200 rpm at 37°C (DA1877) or at 30°C (HGB2511). The bacteria from the overnight culture were pelleted by centrifugation (7745 rcf, 20 minutes) at 4°C, the supernatant was removed, and the pellet was resuspended in S Medium at 10% of the original culture volume. The concentrated bacteria solution was then stored at 4°C until usage.

To prepare the bacteria-based liquid culture medium, the concentrated bacteria solution was warmed up to room temperature and diluted with S Medium to create the different concentrations of the medium used in this study. We do not expect either bacterium to have significant growth in S medium.

**Bacterial-based liquid culture and analyses**

In the liquid culture described in this study, worms were cultured in 24-well tissue culture plates incubated at 25°C on an orbital shaker platform moving at 60 rpm. Inside the incubator, the plates were placed in a plastic shoebox along with wet paper towels that were wetted regularly to maintain humidity. Each well contained 1 mL of culture medium.

To initiate the culture, gravid *S. hermaphroditum* adults grown on *Comamonas/* EPM plates were harvested to obtain embryos based on the method described in Rodak et al. (2024). Briefly, gravid adults were collected from the plates using M9 buffer into a 15 mL centrifuge tube. The worms were then pelleted by centrifugation to remove the supernatant. The worm pellet was then resuspended with water to obtain a total volume of 3.5 mL. 0.5 mL of 5 M NaOH and 1 mL of household bleach (8% available chlorine) were then added to the solution for a total volume of 5 mL. The solution was mixed by shaking and allowed to react for about 5 minutes, by which time most tissues other than the embryos should be visibly dissolved. In our experience, longer reactions substantially decreased the viability of the embryos and should preferably be avoided. The embryos were collected by centrifugation and washed 3 times, each with 10 mL of M9 buffer. After the final wash, the embryos were again pelleted and resuspended in a small volume of M9 before being transferred to liquid cultures. To estimate the number of P _0_ worms, 100 µL of liquid cultures were transferred onto a solid medium DA1877/ EPM plates the day following the initiation of the culture, animals were counted and removed. The plates were checked again the next day for worms that were missed. Six wells were sampled, and the average was calculated. In continuous culture assay, 1 mL cultures containing 20% *C. aquatica* suspension were resupplied daily by adding 25µL, 50µL, or 100µL of concentrated *C. aquatica* to the original culture.

In Fig. 1B and C, the presence of F _1_ progeny was determined by the simultaneous presence of both gravid P _0_ adults and newly hatched F _1_ larvae. The identification of infective juveniles (IJs) was based on features common to the IJ or dauer stages of *Steinernema* and some other nematode species, including slim body shape, sealed mouth, and the presence of an unecdysed J2 cuticle. The observations were performed using a Zeiss Imager Z2 microscope, in which 10 µL of liquid *Steinernema* culture was mounted on a 2% agarose pad, with 1 µL of 100 mM levamisole added to immobilize the animals. Images used in Fig. 1A and 1F were obtained through an Axiocam 506 mono and Zen 2 Blue software using the same configurations. In Fig. 1D and E, worms were sampled every 24 hours by transferring a small portion of the culture onto empty NGM (nematode growth media) (Brenner 1974) plates, where the images of worms were captured using an M2Bio hybrid stereo-compound fluorescence microscope. Worm length was measured using ImageJ (Fiji) (Schindelin et al. 2012) as described in Rodak et al. (2024).
